# Operative ubiquitin-specific protease 22 deubiquitination confers a more invasive phenotype to cholangiocarcinoma

**DOI:** 10.1038/s41419-021-03940-0

**Published:** 2021-07-05

**Authors:** Yu Tian, Bo Tang, Chengye Wang, Yan Wang, Jiakai Mao, Yifan Yao, Zhenming Gao, Rui Liang, Mingliang Ye, Shijie Cai, Liming Wang

**Affiliations:** 1 Division of Hepatobiliary and Pancreatic Surgery, Department of Surgery, The Second Hospital of Dalian Medical University, Dalian, Liaoning PR China; 2 Division of Vascular Surgery, Department of Surgery, The Second Hospital of Dalian Medical University, Dalian, Liaoning PR China; 3 National Key Laboratory of Separation Sciences for Analytical Chemistry, Dalian Institute of Chemical Physics, Chinese Academy of Sciences, Dalian, PR China; 4 Department of Health Sciences, Hiroshima Shudo University, Hiroshima, Japan; 5 Nuffield Division of Clinical Laboratory Sciences, Radcliffe Department of Medicine, John Radcliffe Hospital, University of Oxford, Oxford, UK

**Keywords:** Mechanisms of disease, Preclinical research

## Abstract

Oncogenic ubiquitin-specific protease 22 (USP22) is implicated in a variety of tumours; however, evidence of its role and underlying molecular mechanisms in cholangiocarcinoma (CCA) development remains unknown. We collected paired tumour and adjacent non-tumour tissues from 57 intrahepatic CCA (iCCA) patients and evaluated levels of the USP22 gene and protein by qPCR and immunohistochemistry. Both the mRNA and protein were significantly upregulated, correlated with the malignant invasion and worse OS of iCCA. In cell cultures, USP22 overexpression increased CCA cell proliferation and mobility, and induced epithelial-to-mesenchymal transition (EMT). Upon an interaction, USP22 deubiquitinated and stabilized sirtuin-1 (SIRT1), in conjunction with Akt/ERK activation. In implantation xenografts, USP22 overexpression stimulated tumour growth and metastasis to the lungs of mice. Conversely, the knockdown by USP22 shRNA attenuated the tumour growth and invasiveness in vitro and in vivo. Furthermore, SIRT1 overexpression reversed the USP22 functional deficiency, while the knockdown acetylated TGF-β-activated kinase 1 (TAK1) and Akt. Our present study defines USP22 as a poor prognostic predictor in iCCA that cooperates with SIRT1 and facilitates tumour development.

## Introduction

Cholangiocarcinoma (CCA) is a rare highly malignant bile duct tumour that is classified into intrahepatic CCA (iCCA) and extrahepatic CCA (eCCA) subtypes. The overall incidence of CAA includes 15% of primary liver cancers, the second most common type of primary liver cancer [1, 2]. The incidence of iCCA has increased worldwide over the last few decades [3], there is an unmet need to identify molecular mechanisms that drive this malignancy.

Ubiquitination is one of the main posttranslational modification mechanisms by which regulating protein activation/inactivation, DNA repair, gene regulation and signal transduction [4]. Conjugating ubiquitin to specific proteins through monoubiquitylation or polyubiquitylation is one of the most common posttranslational modifications for regulating protein expression [5, 6]. This process is initiated by ubiquitin-activating enzymes (E1s), followed by ubiquitin-conjugating enzymes (E2s) and ubiquitin ligases (E3s), to modify the targeted proteins [7].

Opposing these enzyme activities, deubiquitination by deubiquitinases (DUBs) removes ubiquitin from the ubiquitinated proteins to maintain physiological homeostasis [8]. So far more than one hundred DUBs have been identified in humans; among them, ubiquitin-specific peptidases (USP) are the largest family [5]. USP22 is one of the subtypes. Under normal physiological conditions, USP22 controls cell cycle progression, protein degradation and embryonic stem cell differentiation [9–12]. By contrast, USP22-associated ubiquitination dysregulation has been implicated in many diseases including tumours [9, 13–18].

USP22 deubiquitylase is a subunit of the transcriptional regulatory histone acetylation complex Spt-Ada-Gcn5 acetyltransferase (SAGA), deubiquitylating the core histone H2B through monoubiquitination by catalysing the removal of ubiquitin from oncoproteins [10, 19]. The induction by USP22 promotes tumour invasion and epithelial-to-mesenchymal transition (EMT), leading to downregulation of E-cadherin and upregulation of N-cadherin, vimentin and matrix metalloproteinases (MMP) [14]. The alterations of these molecules often prevail in malignant lesions. Despite the key role USP22 plays in many types of tumours, evidence for its involvement in CCA development is completely absent and its dichotomous association with different types of tumours—high USP22 expression stimulates breast tumour growth [20], whereas impedes colorectal cancer development [21].

One known substrate of USP22 is sirtuin-1 (SIRT1), a member of the highly conservative mammalian sirtuins family [22–24]. SIRT1 is a highly conserved mammalian NAD^+^-dependent histone deacetylase, acting as a key metabolic sensor that modulates EMT in tumours [25, 26]. By association with USP22 in the SAGA complex, SIRT1 targets protein deacetylation on the structures of their histones or non-histones to facilitate tumour progression [11, 15, 27, 28]. However, whether SIRT1 is pro- or anti-tumour growth, particularly its involvement in CCA associated with USP22, remains unknown.

The purposes of this study were therefore to determine the clinical significance of USP22 expression in iCCA, to evaluate the role of USP22 in controlling tumour growth in vitro and in vivo, and to study the underlying molecular mechanisms by which USP22 operates linking SIRT1 for the epigenetic deubiquitination and deacetylation to CCA progression.

## Materials and methods

### Patients

The iCCA and adjacent non-tumour tissues (2 cm away from the tumours) were collected from patients (*n* = 57) who underwent surgical treatment from October 2009 to October 2015 at the Department of Hepatobiliary and Pancreatic Surgery of the Second Hospital of Dalian Medical University. Tumour staging was classified according to the International Union Against Cancer to the tumour-node-metastasis (TNM) [29]. The median of follow-up period was 16.7 months. The use of the tissues was approved by our Hospital Ethics Board (No. 2016007). Written informed consent was obtained from patients before the surgery.

### Reagents

The Cell Counting Kit-8 (CCK-8), Annexin V-FITC Apoptosis Detection Kit and Cell Cycle Detection Kit were purchased from KeyGen Biotech (Nanjing, China). MG132 and cyclohexymide (CHX) were from Sigma (St. Louis, USA).

### Cell culture and transfection

Human CCA cell lines RBE and HCCC-9810 (HCCC) were purchased from the Type Culture Collection of the Chinese Academy of Sciences (Shanghai, China), Huh28, HuCCT1, QBC939 were provided by 3D Medicines (Shanghai, China). All cell lines were authenticated by the short tandem repeat (STR). Cells were cultured in RPMI-1640 medium (Gibco, USA) supplemented with 10% fetal bovine serum (FBS, Gibco, USA), 100 µg/ml penicillin and 100 µg/ml streptomycin (Invitrogen, USA) in a 5% CO_2_ atmosphere at 37 °C. All the cell lines were authenticated by STR profiling. siRNAs were used for USP22 transiently silencing. They were transfected with Lipofectamine 2000 reagent (Invitrogen, USA) according to the manufacturer’s instructions. Lentivirus-mediated shRNAs knockdown or gene overexpression of USP22 or SIRT1 were used to construct stable cell lines.

### Cell proliferation and colony formation

To measure cell proliferation, 3 × 10^3^ cells/well were grown in 96-well plates and CCK-8 kit was used to quantify them. For colony formation assay, 1 × 10^3^ cells/well were seeded in six-well plates. After 14 days, colonies were fixed with 4% PFA and stained with 1% crystal violet, in which visible colonies were counted (>50 cells/colony).

### Migration and invasion

In terms of migration assay, 2 × 10^5^ cells in 200 μl of serum-free medium were plated in an 8μm pore size transwell inserts and grown in 300 μl of DMEM with 10% fatal bovine serum. For invasion assay, the same culture conditions were applied for the experiment but the insert was precoated with 50 μl of 1 mg/ml Matrigel (BD, Franklin Lakes, NJ, USA). After 24 h, cells migrated to the lower surface of the membrane, after which they were fixed with formalin and stained with 1% crystal violet. Five random fields were selected for the quantification at ×10 magnification.

### Western blot

Samples were lysed in RIPA buffer and the lysate protein was determined by BCA assay kit. The protein lysates were separated by 10% SDS-PAGE and transferred onto PVDF membranes. After blocking, their blots were incubated with primary and secondary antibodies, and the bands were visualized by chemiluminescence.

### Co-Immunoprecipitation (Co-IP)

Co-IP was done using the Pierce^TM^ co-immunoprecipitation kit following the manufacturer’s protocol. Briefly, cell lysates were prepared and the primary antibody was first immobilized for 2 h using Amino Link Plus coupling resin. The resin was then washed and incubated with cell lysates overnight. After protein elution, samples were analysed by immunoblotting.

### Immunohistochemistry (IHC)

Tissues were fixed, embedded in paraffin wax and sectioned and prepared for haematoxylin and eosin (H&E) or IHC staining [30]. The intensity of the staining was determined and scored as described previously [31]. These scores were determined independently by two experienced pathologists in a blinded manner.

### Immunofluorescence staining

Cells were fixed with 4% paraformaldehyde, permeabilized with 0.1% Triton X-100, and blocked with 5% bovine serum albumin (BSA). Followed by the primary and second antibodies sequentially and DAPI nuclei staining, cells were imaged under laser-scanning confocal microscopy (Zeiss ISM510 META).

### Flow cytometry analysis (FACS)

For apoptosis analysis, 1 × 10^5^ cells were examined using an Annexin V-FITC Apoptosis Detection kit according to the manufacturer’s instructions.

For analysis of E-caderin or Vimentin, 1 × 10^7^ cells were seeded in six-well plates. After 48 h, they were detached with an enzyme-free cell dissociation buffer, subsequently fixed with fixation permeabilization solution before proceeding the step with BD perm/wash buffer. Samples were incubated with their respective primary and secondary antibodies and analysed by FACS Calibur (BD Biosciences) analyzer.

### Xenograft and metastasis in vivo

Five–week old BALB/c nude male mice were purchased from Beijing Vital River Laboratory Animal Technology Co., Ltd. China. They were healthy and their body weight was 25 ± 15 g. Under specific pathogen-free conditions, they were housed in plastic cages, with wood chips, which were changed every week, and each cage was kept to five heads without mixing gender. The dark/light cycle is 12/12 h, and room temperature is kept at 20 ± 2 °C. All mice were allowed free access to water and sterilised normal chow. All the mice were randomized. The experimental protocols were made in accordance with The Regulations for Animal Experiments in Dalian Medical University, which states replacement, refinement or reduction (the 3Rs), and approved by Dalian Medical University Animal Care and Use Committee. Prior to or during surgical procedures, mice were anaesthetised with isoflurane using an isoflurane vaporizer (Kent Scientific Corporatio, USA), which is considered as a simple, safe and non-invasive method. For xenografts or metastasis to the lungs, 1 × 10^7^ cells were injected subcutaneously into individual nude mice or 1 × 10^6^ cells were injected intravenously into the tail vein of mice (5 mice per group), respectively. The tumour volumes were measured weekly and calculated according to the formula length × width^2^/2. There were no significant unexpected adverse events. When the tumour reached the permitted maximum volume, or mice showed any signs of too much illness or stress, or 6 weeks after the tail vein injection of tumour cells, mice were humanely culled by cervical dislocation after anaesthesia with isoflurane inhalation following The Regulations for Animal Experiments in Dalian Medical University. The entire tumour or the lung tissues were fixed and sectioned for H&E staining. All the animal experiments were performed and repeated in the Animal Experimental Centre of Dalian Medical University. No blinding to the group allocation during the experiment was done.

### TCGA data analysis

The CHOL RNA-seq (HTSeq–Counts) were retrieved from https://portal.gdc.cancer.gov. Differential expression was analysed on the count matrix with DESeq2 package in R software. An adjusted *p*-value (Padj) < 0.05 was considered statistically significant. The normalized counts by the sequencing depth and adding a pseudocount of 1/2 were extracted with the DESeq2 software package, using the function plotCounts with the argument returnData set to TRUE. Log_2_ (normalized count + 1) was used for log-scale visualization of USP22 expression in tumours and adjacent non-tumour tissues.

### Statistical analysis

All experiments were conducted three times. The data of different experimental groups meet normal distribution and the variance is similar between the groups. Values were presented as the mean ± standard deviation (SD). Student’s *t*-test (two-sided), one-way ANOVA and chi-square (*χ*^2^) analysis were performed to calculate variance. Survival analysis was conducted using the Kaplan–Meier and the log-rank test was used to compare survival rates between groups. *p* < 0.05 indicated statistical significance.

## Results

### Upregulation of USP22 in patients with CCA

**Fig. 1:**
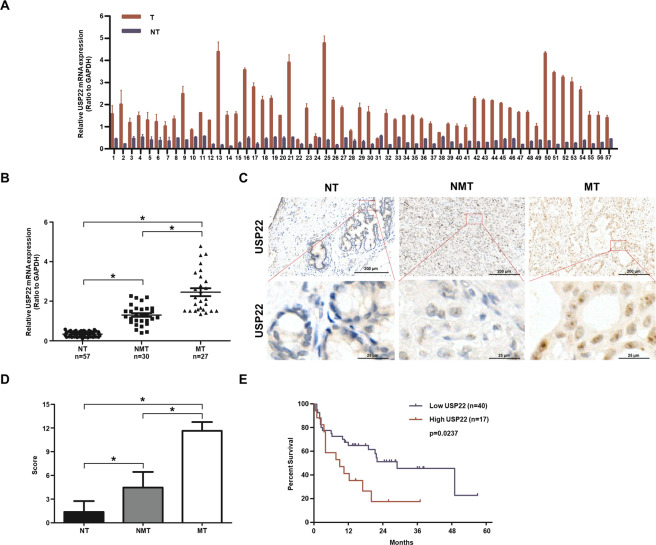
Worse prognostic significance of USP22 overexpression in iCCA. **A** RNAs from paired iCCA and adjacent normal tissues from individual patients (*n* = 57) were respectively extracted and quantified for USP22 mRNA expression by qPCR. **B** Levels of USP22 mRNA were higher in iCCA than the adjacent normal tissues (NT) (*n* = 57) (*p < 0.05), particularly those with metastatic tumours (MT) (*n* = 27) vs non-metastatic tumours (NMT) (*n* = 30) (*p* < 0.05). **C** IHC for USP22 in iCCA and the adjacent normal tissue sections were performed, showing the nuclear localisation of UPS22 (scale bar = 100 µM or 200 µM). **D** High scores of USP22 expression were significantly elevated in iCCA tissues, particularly in MT (*n* = 27) vs NT (*n* = 57) or NMT (*n* = 30) (*p < 0.05). **E** Kaplan–Meier survival curves showed a significant correlation of the high USP22 group (*n* = 16) with worse overall survival than the low expression group (*n* = 41) (*p < 0.05).

We collected 57 paired iCCA and adjacent normal tissues and analysed their USP22 mRNA expression by qPCR, and showed that USP22 was elevated across all tumour tissues (Fig. 1A), particularly those with invasive lesions (*p* < 0.05) (Fig. 1B). By comparing 36 non-paired CCA (30 iCCA and 6 eCCA) with the non-tumour tissues (https://portal.gdc.cancer.gov/), we showed that the USP22 mRNA was significantly upregulated in CCA (*p* < 0.05) (Supplementary Fig. 1A and 1B), but had no significant correlation with copy numbers of USP22 with either shallow deletion, DNA diploid, or amplification (Supplementary Fig. 1C), hinting the non-genetic regulatory role of USP22 in CCA.

We showed that USP22 protein expression by IHC was consistently upregulated in CCA, present predominantly in the tumour cell nucleus but rarely in any other subtypes of cells (Fig. 1C). The expression was significantly upregulated in tumours (*p* < 0.05) (Fig. 1D). Our correlation analysis based on high IHC scores showed that USP22 was significantly upregulated in CCA (*p* < 0.05) (Fig. 1D), mainly in patients with a larger tumour, microvascular invasion and lymph node metastasis during a complete 57-month follow-up with the median period of 16.7 months (Table 1). Importantly, those patients with high levels of UPS22 protein expression had a worse overall survival (OS) (*p* < 0.05) (Fig. 1E), revealing that USP22 may be a poor prognostic marker to predict CCA progression.

**Table 1:** Immunohistochemical analysis of USP22 expression with clinicopathology of CCA.

Tumour characteristics	USP22 protein expression		Total	*p*
	Low	High		
Age (year)				
≤50	7 (47%)	8 (53%)	15	0.389
>50	25 (60%)	17 (40%)	42	
Sex				
Male	27 (55%)	22 (45%)	49	0.696
Female	5 (63%)	3 (37%)	3	
γ-GT (U/L)				
≤54	19 (56%)	15 (44%)	34	0.962
>54	13 (57%)	10 (43%)	23	
Liver cirrhosis				
No	2 (67%)	1 (33%)	3	0.706
Yes	30 (56%)	24 (44%)	54	
Tumor diameter				
≤5	26 (67%)	13 (33%)	39	0.018
>5	6 (33%)	12 (67%)	18	
Microvascular invasion				
Absent	22 (73%)	8 (27%)	30	0.006
Present	10 (37%)	17 (63%)	27	
Tumor encapsulation				
Complete	14 (67%)	7 (37%)	21	0.221
None	18 (50%)	18 (50%)	36	
Tumor differentiation				
I + II	26 (68%)	12 (32%)	38	0.080
III + IV	6 (32%)	13 (68%)	19	
Lymph node metastasis				
No	20 (77%)	6 (23%)	26	0.004
Yes	12 (39%)	19 (61%)	31	
TNM stage				
I	17 (57%)	13 (43%)	30	0.933
II + III	15 (56%)	12 (44%)	27	

Correlation of USP22 protein expression with clinicopathology in 57 CCA in 2nd Hospital of Dalian Medical University, China.

*γ-GT* γ-glutamyl transferase, *TNM* tumour-nodes metastasis.

*p*-value was calculated using the Pearson *χ*^2^ test, and *p* < 0.05 was considered a statistically significant difference.

### USP22 overexpression stimulates cell proliferation, migration/invasion, and induces EMT in CCA in vitro

Our immunoblotting analysis showed that USP22 was expressed relatively higher in CCA cell lines of RBE, QBC939 and HuCCT1 than in HCCC and Huh28 (Supplementary Fig. 2A). By transiently transfecting 3 different pairs of siRNAs into RBE confirmed that the second pair gave rise to the better knockdown of the USP22 gene than the other 2 pairs (Supplementary Fig. 2B). Accordingly, we established RBE and QBC939 lines with USP22 stably knockdown by shRNA and designated them as RBE-shSP22 and QBC939-shUSP22, and their controls as RBE-shControl and QBC939-shControl (Supplementary Fig. 2C). By the lentiviral gene transfers of USP22 or empty vector (EV), we established lines of HCCC-USP22 and Huh28-USP22, and the respective HCCC-EV and Huh28-EV controls (Supplementary Fig. 2D).

**Fig. 2:**
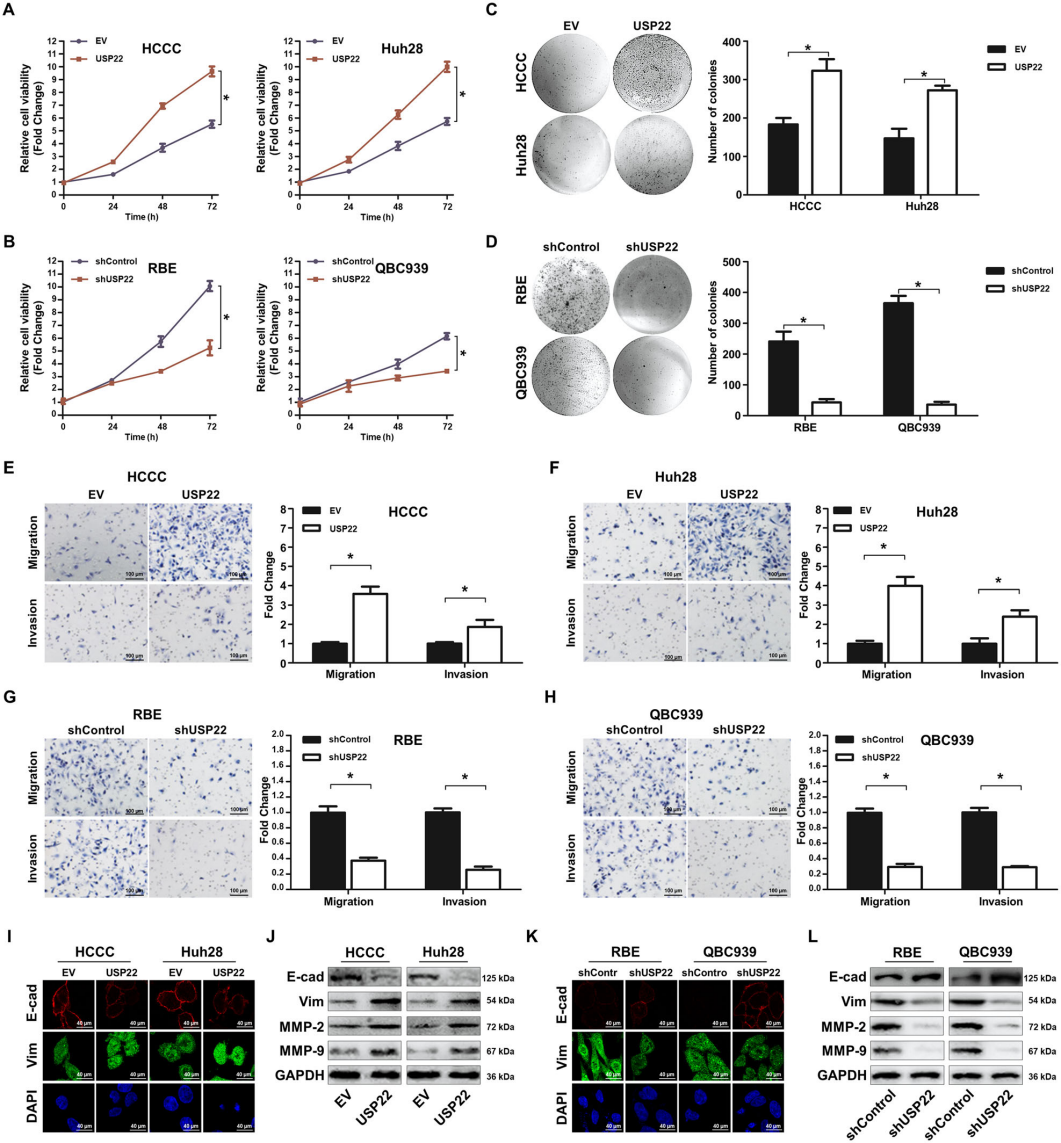
USP22 promotes cell proliferation, migration and invasion, and induces CCA EMT in vitro. **A**–**B** HCCC-USP22, Huh28-USP22, RBE-shUSP22, QBC939-shUSP22 and their controls were cultured and their proliferation was evaluated once a day for 3 consecutive days by CCK8 assay. **C**–**D** Each cell line was seeded into six-well plates with 5,000 cells/well. Colonies formed after 14 days were fixed and stained for quantification. **E**–**H** Cells were plated in cell inserts with 1 × 10^4^ cells/insert (8 µm pore size) with/without matrigel for invasion or migration assay, respectively. After 24 h, five images per insert were randomly taken and quantified by ImageJ. **I**–**J** HCCC-USP22, Huh28-USP22 and their controls were cultured for 48 h. The cells were fixed and immunostained with antibodies to E-cadherin (red) and Vimentin (green), and with DAPI for DNA. Cell lysates from these cell lines were also prepared for immunoblotting analysis with antibodies to E-cadherin, Vimentin, MMP2, MMP9 and GAPDH. **K**–**L** RBE-shUSP22, QBC939-shUSP22 and their controls were cultured for 48 h. The cells were fixed and immunostained with antibodies to E-cadherin (red) and Vimentin (green), and with DAPI for DNA. All data are presented as the mean ± SD (*p < 0.05, *n* = 3).

USP22 overexpression increased cell proliferation (*p* < 0.05) (Fig. 2A) and colony formation in HCCC-USP22 or Huh28-USP22 compared with HCCC-EV or Huh28-EV controls, respectively (*p* < 0.05) (Fig. 2C). USP22 silencing decreased cell proliferation and clonogenesis by 3-folds in both RBE-shSP22 and QBC939-shUSP22 in comparison to RBE-shControl or QBC939-shControl (*p* < 0.05) (Figs. 2B and D).

Furthermore, USP22 overexpression enhanced HCCC and Huh28 cell migration and invasion by ~2–4 folds (*p* < 0.05) (Fig. 2E and F), while USP22 silencing reduced RBE and QBC939 cell mobility by more than 70% (*p* < 0.05) (Fig. 2G and H).

Immunofluorescence staining revealed that USP22 overexpression downregulated the cellular membrane localised E-cadherin and upregulated vimentin in HCCC and Huh28 when they were compared with HCCC-EV or Huh28-EV controls (Fig. 2I). Similar results were reproduced by immunoblotting analysis (Fig. 2J) and the quantitative assay of FACS (Supplementary Fig. 3A–3F) (*p* < 0.05). USP22 enhanced both MMP2/9 expression, the tumour invasive markers (Fig. 2J). The opposite was true when the USP22 gene was knocked down in RBE or QBC939 when collated with the respective controls (Figs. 2K and L, Supplementary Fig. 3G-3L).

### USP22 overexpression induces tumour growth and the metastasis to the lungs of mice in vivo

**Fig. 3:**
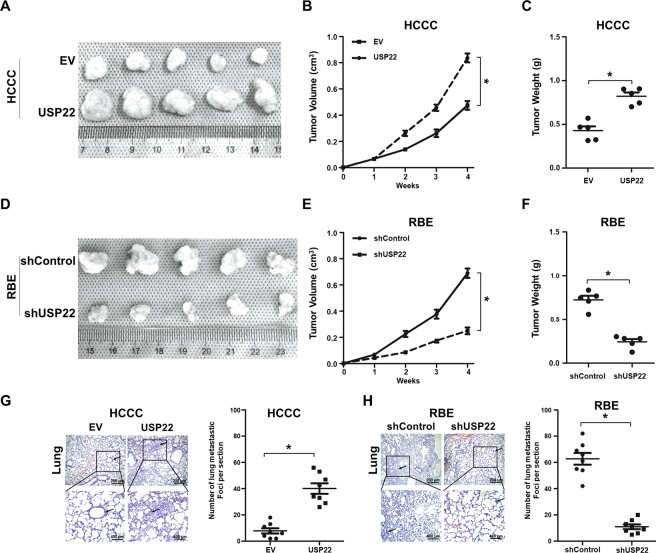
USP22 promotes CCA tumorigenesis and metastasis in vivo. **A**–**F** Each of 1 × 10^7^ HCCC-USP22, HCCC-EV, RBE-shUSP22 or RBE-shControl cells were subcutaneously injected into the flanks of 5-week-old nude mice. Tumour volume was monitored once a week; when they reached the maximum permitted volumes, mice were sacrificed, and tumours were weighted (*p < 0.05, *n* = 5). **G**–**H** Each of 1 × 10^7^ HCCC-USP22, HCCC-EV, RBE-shUSP22 or RBE-shControl cells were injected into the tail veins of nude mice. After 6 weeks, animals were sacrificed, and their lungs were harvested and fixed for H&E staining. Numbers of metastatic foci were quantified. Data are shown as the mean ± SD (*p < 0.05, *n* = 8). Representative images of metastatic foci in sections of the lungs.

We subcutaneously injected cell lines of USP22-overexpressing HCCC-USP22 or USP22-silenced RBE-shSP22, and their control counterparts into the nude mice. The sizes and weights of HCCC-USP22 tumours were twofolds greater than the HCCC-EV controls (*p* < 0.05) (Fig. 3A–C). However, RBE-shSP22 tumour sizes and weights were only half of the RBE-shControl (Fig. 3D–F).

After a 6-week period of the injection of HCCC-USP22 or HCCC-EV control into the tail veins of the mice, we harvested the lungs and stained the tissue sections with H&E and showed number of the metastatic foci in HCCC-USP22 were twice more than in HCCC-EV (*p* < 0.05) (Fig. 3G). Conversely, the total number of RBE-shUSP22 metastatic foci were only 1/3 of RBE-shControl (*p* < 0.05) (Fig. 3H).

### Association of USP22 expression with SIRT1 in CCA

**Fig. 4:**
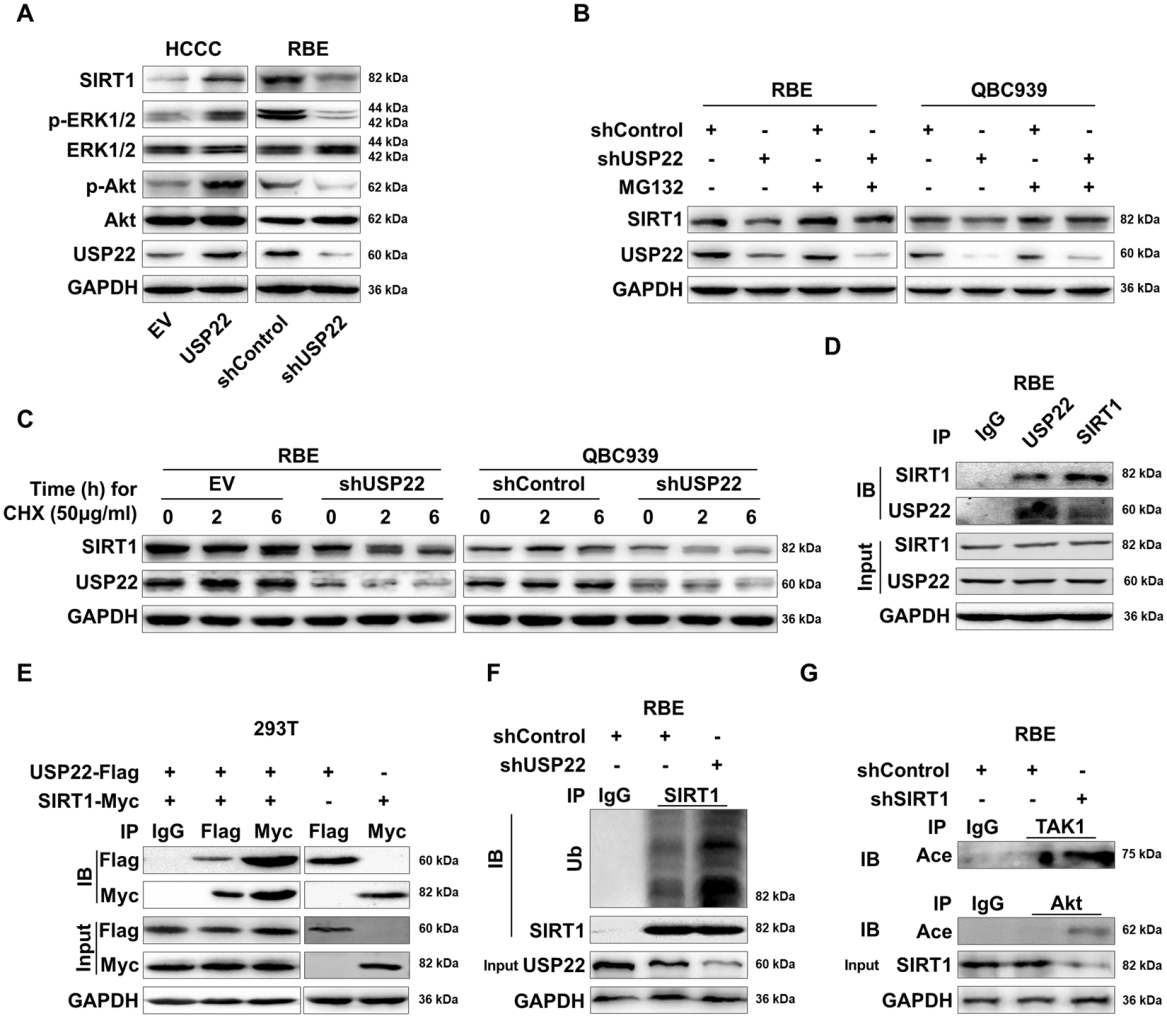
USP22 stabilizes SIRT1 and regulates phosphorylation of Akt and ERK1/2. **A** HCCC-USP22, HCCC-shControl, RBE-shUSP22 or RBE-shControl cells were prepared for western blot analysis with antibodies to SIRT1, phosph-ERK1/2, ERK1/2, phosph-Akt (Tyr 473), Akt and GAPDH. **B** RBEshUSP22/RBE-shControl or QBC939-shUSP22/QBC939-shControl were cultured ± MG132 (10 μM) for 6 h. Cells were lysed and prepared for immunoblotting analysis with antibodies to SIRT1, USP22 and GAPDH. **C** RBE-shUSP22/RBE-shControl or QBC939-shUSP22/QBC939-shControl were cultured ± cycloheximide (CHX) 50 μg/ml and harvested at the time points of 2 and 6 h, and cells were lysed for immunoblotting analysis with antibodies to SIRT1 and USP22. **D** Cell lysates from RBE-shControl and RBE-shUSP22 were prepared for reciprocal pull-down and immunoblotting analysis with antibodies to IgG, USP22 and SIRT1. **E** 293 T cells were grown and cotransfected with Flag-tagged USP22 and Myc-SIRT1, and the cell lysates were prepared for immunoprecipitation and immunoblotting analysis with antibodies to IgG, Flag and Myc. **F**, **G** RBE-shControl and RBE-shUSP22 cells were cultured and cell lysates were prepared for immunoprecipitation with antibodies to IgG, SIRT1, TAK1 or Akt. They were analysed by immunoblotting with antibodies to ubiquitin, SIRT1, USP22, pan-acetylation, TAK1, Akt, and GAPDH. All data shown are representative of three independent experiments.

By immunoblotting analysis, we showed that USP22 overexpression upregulated SIRT1, with increased ERK1/2 and Akt phosphorylation in HCCC-USP22 compared their basal levels with HCCC-EV (Fig. 4A). In contrast, USP22-silenced RBE gave rise to an opposite result (Fig. 4A), suggesting the regulation of SIRT1 and ERK/Akt phosphorylation by USP22. Essentially, USP22 silencing decreased a fraction of SIRT1 expression in RBE and QBC939 (Fig. 4B). Treatment of them with MG132, a proteasome inhibitor, retained the levels of SIRT1 in RBE-shSP22 or QBC939-shUSP22, which were comparable to the respective RBE-shControl or QBC939-shControl (Fig. 4B). Repeatedly incubating USP22-silenced RBE and QBC939 or the respective controls with cycloheximide (CHX) to block protein translation, we showed downregulation of SIRT1 in a trend of time-independent fashion. At the same time, the level of SIRT1 expression remained constant in RBE-shControl or QBC939-shControl (Fig. 4C), suggesting dependency of SIRT1 expression on USP22 in CCA.

To determine the physical interactions in CCA, we performed Co-IP by immunoprecipitating the endogenous SIRT1 or USP22 proteins and showed the reciprocal immunoprecipitation of them by the respective antibodies from RBE-shUSP22 or RBE-shControl cell lysates, but not by the IgG control (Fig. 4D). Furthermore, we evaluated their interactions by transiently transfecting Flag-tagged SIRT1 or Myc-tagged USP22 alone or co-transfecting them into 293T cells. After 48 h, their cell lysates were prepared for Co-IP and demonstrated the reciprocal immunoprecipitation by the respective antibodies to Flag or Myc (Fig. 4E), reinforcing the presence of the complex formation of SIRT1 and USP22.

To investigate if SIRT1 degradation was ubiquitination-dependent when USP22 was silenced, we repeated the pull-down to detect the endogenous SIRT1 in RBE cell lysates with an antibody to SIRT1 and the IgG isotype control. After immunoblotting with antibodies to ubiquitin, SIRT1, USP22 and GAPDH, we showed that SIRT1 was ubiquitinated and accumulated in the USP22-silenced RBE but not in the control (SIRT1 as the loading control) (Fig. 4F). These findings demonstrate the unbiquitination-mediated SIRT1 degradation when USP22 is downregulated in RBE.

Next, we evaluated the effect of SIRT1 on oncoprotein TGF-β-activated kinase 1 (TAK1) and Akt acetylation and also conducted immunoprecipitation with antibodies to TAK1 and Akt, followed by immunoblotting with antibodies to pan-acetylation, TAK1, Akt, SIRT1 and GAPDH. We showed increased acetylated TAK1 or Akt in SIRT1-silenced RBE (Fig. 4G), suggesting that TAK1 and Akt acetylation depends on SIRT1 in RBE (Supplementary Fig. 4). The same outcomes occurred in USP22-silenced in RBE. Moreover, USP22 overexpression in HCCC cells downregulated acetylation of TAK1 and Akt (Supplementary Fig. 4).

Collectively, we show that USP22 and SIRT1 are part of a protein complex and USP22 regulates SIRT1 deubiquitination. Both USP22 and SIRT1 epigenetically modifies TAK1 and Akt, involving deacetylation a previous unidentified observation in CCA growth.

### Functional association of USP22 with SIRT1 in vitro and in vivo

**Fig. 5:**
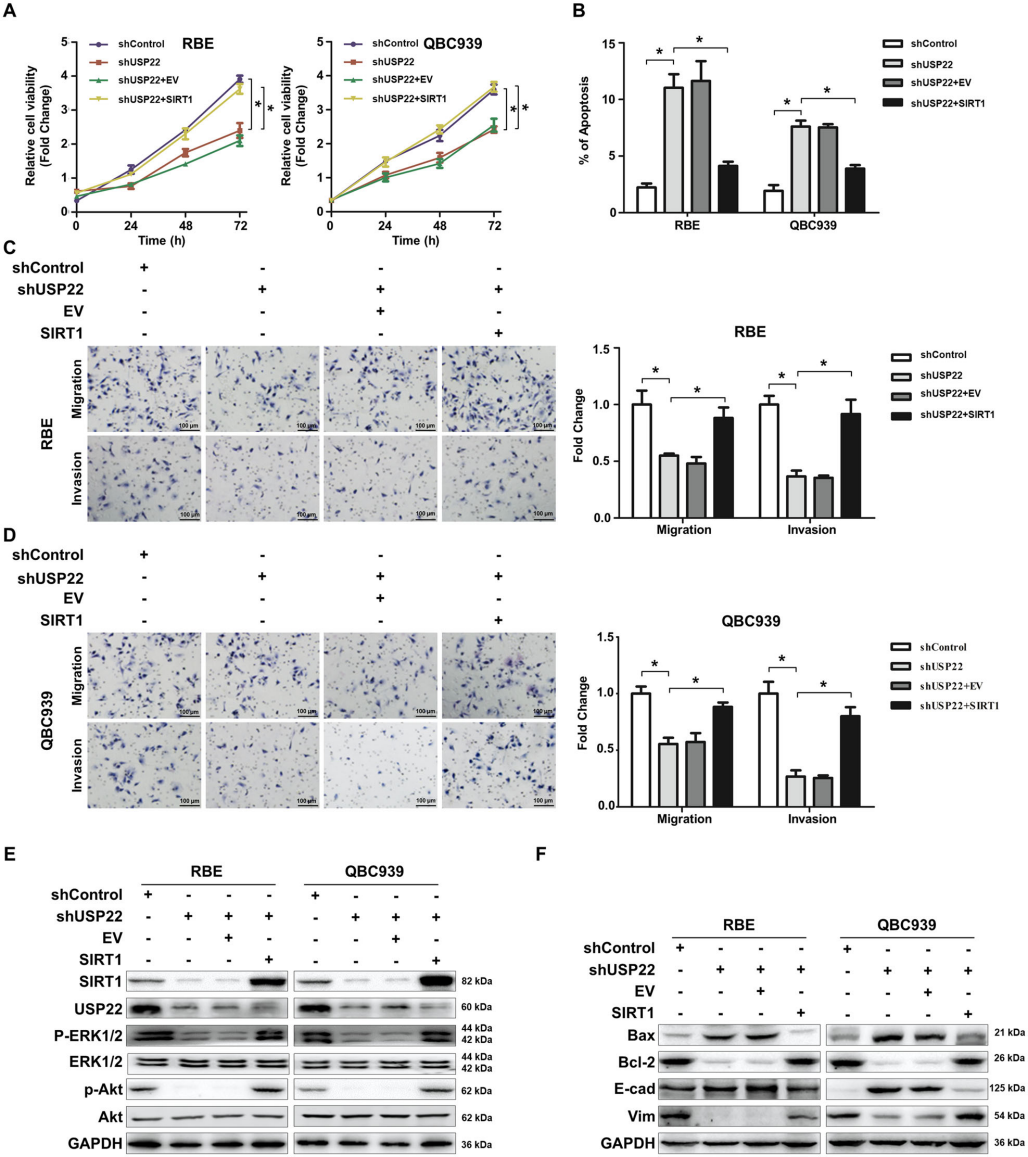
SIRT1 restores CCA growth and metastasis independently of USP22 in vitro. **A**–**D** Cell lines of RBE-shUSP22, RBE-shControl, RBE-shUSP22+EV and RBE-shUSP22 + SIRT1 or QBC939-shUSP22, QBC939-shControl, QBC939-shUSP22 + SIRT1 and QBC939-shUSP22+EV were grown for the assay of cell proliferation by CCK-8 (**A**). They were also prepared for assays of apoptosis by FACS (**B**), and transwell migration/invasion as shown in Fig. 2**(C**–**D)**. All data shown are presented as the mean ± SD (*p < 0.05, *n* = 3). **E**–**F** Cell lines mentioned above were also cultured and the lysates were prepared for immunoblotting analysis with antibodies to SIRT1, USP22, p-Akt and p-ERK1/2, Bax, Bcl-2, E-cadherin, vimentin and GAPDH. All data shown are representative of three independent experiments.

To determine the functional interplays between USP22 and SIRT1, we overexpressed SIRT1 in USP22-silenced RBE or QBC939 (namely RBE-shUSP22 + SIRT1 or QBC939-shUSP22 + SIRT1 and the controls of RBE-shUSP22 + EV or QBC939-shUSP22 + EV). SIRT1 overexpression restored capabilities of USP22-silenced RBE and QBC939 cell proliferation, migration and invasion (*p* < 0.05) (Figs. 5A–D), in conjunction with increased anti-apoptosis in vitro (*p* < 0.05) (Fig. 5B). SIRT1 overexpression also rescued their cell mobility by triggering them to migrate and invade to the extent that was comparable to the abilities of the controls in vitro (Fig. 5C and D).

We showed that SIRT1 overexpression in USP22-silenced RBE and QBC939 phosphorylated ERK1/2 and Akt (Fig. 5E), and decreased pro-apoptotic Bax, with spontaneously increased anti-apoptotic Bcl-2 expression as analysed by immunoblotting (Fig. 5F). SIRT1 overexpression rewrote an EMT programme by upregulating vimentin and downregulating E-cadherin in USP22-silenced RBE and QBC939. In keeping with these immunoblotting analyses, similar results were observed in USP22-silenced RBE and QBC939 from FACS analysis (Supplementary Fig. 5A–F). Thus, our study suggests that SIRT1 regulates CCA survival and EMT induction, but independently USP22.

**Fig. 6:**
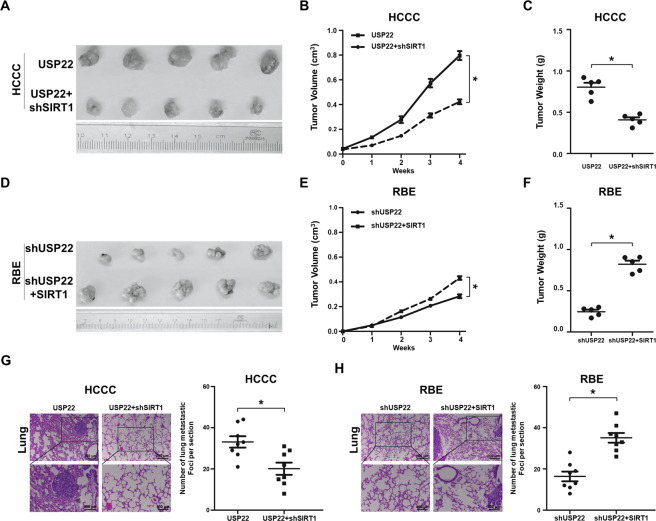
SIRT1 restores CCA growth and induces tumour metastasis to the lungs of mice independently of USP22 in vivo. **A**–**F** Each of 1 × 10^7^ HCCC-USP22 or HCCC-USP22 + shSIRT1, RBE-shUSP22 or RBE-shUSP22 + SIRT1 cells were subcutaneously injected into the flanks of 5-week-old nude mice. Tumour volume was monitored once a week; when they reached the maximum permitted volumes, mice were sacrificed, and tumours were weighted (*p < 0.05, *n* = 5). **G-H**. Each of 1 × 10^7^ HCCC-USP22 or HCCC-USP22 + shSIRT1, RBE-shUSP22 or RBE-shUSP22 + SIRT1 cells were injected into the tail veins of nude mice. After 6 weeks, animals were sacrificed, and their lungs were harvested and fixed for H&E staining. Numbers of metastatic foci were quantified. Data are shown as the mean ± SD (*p < 0.05, *n* = 8). Representative images of metastatic foci in sections of the lungs.

We validated these in vitro findings in vivo by subcutaneously injecting SIRT1-silenced USP22-overexpressing HCCC (namely HCCC-USP22 + shSIRT1) and HCCC-USP22 control, or SIRT1 overexpression in USP22-silenced RBE (namely RBE-shUSP22 + SIRT1) and RBE-USP22 control. SIRT1 knockdown inhibited USP22-induced HCCC tumour growth (Fig. 6A), and the sizes and weights of these tumours were almost half of the HCCC-USP22 (*p* < 0.05) (Fig. 6B and C). Overexpression of SIRT1 stimulated RBE-shUSP22 tumour xenograft growth—the sizes and the weights of the RBE-shUSP22 + SIRT1 tumours were twofold greater than the RBE-shUSP22 control (*p* < 0.05) (Fig. 6D, E and F).

By injecting these cell lines into mouse tail veins, we detected that decreased the total number of metastatic foci in the mouse lungs of SIRT1-silenced HCCC-USP22, which were half of the HCCC-USP22 control (*p* < 0.05) (Fig. 6G). Conversely, overexpression of SIRT1 in USP22-silenced RBE generated twofold greater of the metastatic foci than that of the RBE-shUSP22 control mouse lungs (*p* < 0.05) (Fig. 6H).

Essentially, our clinical analysis of H-score for USP22 and SIRT1 in 57 CCA tissue samples demonstrated a positive correlation between them (*p* < 0.05) (Supplementary Table 1). We analysed their relationship at mRNA levels in a TCGA dataset and found that they were not significantly correlated (Supplementary Fig. 6), suggesting their post-translational cooperation for CCA.

## Discussion

Much evidence has shown USP22 is overexpressed in a variety of tumours but if it is protumourigenic posing a contradictory outcomes and investigation of its role in CCA remains completely unknown. In the present study, we showed that both high USP22 gene and protein was significantly correlated with the worse prognosis of iCCA, particularly those with highly invasive lesions. USP22 favoured tumour cell proliferation, migration and invasion in vitro and in vivo. The underpinning protumourigenic USP22 on tumour cells was EMT induction, exhibiting loss of epithelial E-cadherin and gain of mesenchymal vimentin, with upregulation of MMP2/9 invasive markers in CCA cell lines. The USP22 deubiquitination-associated SIRT1 stabilization led to the increased downstream ERK/Akt phosphorylation and anti-apopotic Bcl expression and spontaneously decreased proapopotic Bax expression. SIRT1 overexpression was capable of functionally restoring the genetically knockdown of the USP22 gene in CCA in vitro and in vivo. Furthermore, the knockdown of SIRT1 led to TAK1 and Akt acetylation, linking the role of SIRT1 plays in regulating deacetylation—a previous unreported mechanism in CCA.

USP22 overexpression is correlated with poor clinical outcomes in carcinomas of salivary duct, esophageal squamous cell and liver [32–34]. That is clearly confirmed here and extended by showing that USP22 was an independent poor prognostic predictor for iCCA. Our analysis in a TCGA dataset indicates that there was no significant correlation of the levels of the USP22 mRNA with the mutation of USP22 in the states of either a shallow deletion, diploid or gain/amplification, suggesting posttranslational regulation of CCA by USP22.

Our determination of USP22 in human CCA cell lines in vitro and in vivo showed that USP22 overexpression was tumorigenic, promoting cell growth, mobility and metastasis, and inducing EMT. Conversely, the shRNA knockdown convincingly reserved the USP22 these protumourigenic functions. These results are in lines with several previous reports [35] suggesting that USP22 is a potential oncogenic driver in regulating the development of CCA.

USP22 has been involved in a plethora of physiological and pathophysiological activities, as cell cycle regulation, anti-apoptosis and cancer development [36]. As a component of the deubiquitinating module of the SAGA complex, USP22 deubiquitinates H2B via monouquibination to regulate transcriptional activity and stabilizes SIRT1 for maintaining physiological metabolic homeostasis [28]. We extended that further by showing that USP22 deubiquitination was associated with SIRT1 stabilisation, subsequently transducing Akt and ERK tumour survival signal. A similar process in tumour progression also occurs in many other types of tumours, plausibly involving suppression of p53 transcription to prevent cell death and interact with c-Myc oncogene to maintain CCA progression [9, 12, 36–38].

The question remains however if SIRT1 is a CCA promoter or a suppressor when USP22 is deficient. Our present study showed that ectopic expression of SIRT1 greatly enhanced CCA cell growth and invasion in vitro and in vivo. The opposite was true when the USP22 gene was knocked down in two different CCA cell lines, RBE and QBC939. It activated tumour survival signalling and restored the induction of EMT, accompanied by decreased pro-apoptotic Bax and increased anti-apoptotic Bcl2. This is consistent with the protumourigenic role of SIRT1 in inducing EMT in pancreatic cancer, hepatocellular carcinoma and gastric cancer, but discordant with its EMT inhibition in breast cancer, lung cancer and ovarian cancer [39–44]. Apparently, dual functions of SIRT1 play either protumourigenesis or antitumourignesis, depending on the cellular context of tumour types [45]. SIRT1 may act as a promoter in the context of USP22 for CCA growth.

Furthermore, our present study demonstrates that protumourigenic SIRT1 expression stabilised TAK1 and Akt oncoproteins through deacetylation mediated epigenetic modulation in CCA. TAK1 is a member of the mitogen-activated protein kinase kinase kinase (MAP3K) family that is activated by TGF-β [46]. TAK1 activation promotes cell survival, differentiation, apoptosis, and inflammatory responses [47]. However, TAK1 deacetylation by SIRT1, particularly in the content of tumours, has not been reported previously. Our present study demonstrated that SIRT1 suppression by shRNA increased both TAK1 and Akt acetylation in CCA. Hence the presence of SIRT1 would deacetylate both TAK1 and Akt, and sustain the oncogenic signal cues for CCA development [48–50]. But the exact role of the SIRT1-TAK1-Akt axis in association with epigenetic modification requires a thorough investigation in CCA.

In summary, little is known about the role of USP22 and its relationship with SIRT1 in CCA development. Our present study demonstrates that USP22 is highly expressed in the aggressive iCCA. USP22 stimulates cell proliferation, migration and invasion through EMT induction, by which it deubiquitates and stabilises SIRT1 for CCA development. In conjunction with TAK1 and Akt deaceylation, SIRT1 aggravates CCA interacting with USP22 to epigenetically modulate CCA and facilitate the malignant growth. Thus, their cooperation may pose the synthetic lethality in CCA.

## Supplementary information

Supplementary Figure legends

Supplementary methods

Supplementary Fig. 1

Supplementary Fig. 2

Supplementary Fig. 3

Supplementary Fig. 4

Supplementary Fig. 5

Supplementary Fig. 6

## Data Availability

Code for TCGA data analysis is available from Dr. Yu Tian (dyqnzty@126.com).
