# Supplementary material for: Operative ubiquitin-specific protease 22 deubiquitination confers a more invasive phenotype to cholangiocarcinoma
Source: Cell Death Dis. 2021 Jul 5;12(7):678. doi: 10.1038/s41419-021-03940-0 (PMC8257691; doi:10.1038/s41419-021-03940-0)
Supplement: Supplementary file 1 — Supplementary Figure legends [file 41419_2021_3940_MOESM1_ESM.doc]

**Supplementary Figure 1. Analysis of USP22 mRNA expression in TCGA dataset.**

TCGA-CHOL RNA-seq (HTSeq-Counts) data was downloaded from GDC data portal (<https://portal.gdc.cancer.gov/>) and screened for the differential USP22 expression between tumour and the normal tissues by DESeq2. The USP22 mRNA was significantly higher in CCA than unpaired normal tissues (36 tumour vs 9 non-tumour) **(A)**. The USP22 mRNA also was significantly increased in iCCA compared to paired adjacent tissues (n = 9) **(B)**. Among 36 CCA samples, there was no significant correlation between the USP22 mRNA and the genetic alterations.

**Supplementary Figure 2. Characterisation of basal expression of USP22 and genetically modified USP22 expression CCA cell lines.**

Prior to immunoblotting analysis with antibodies against USP22 or GAPDH, we cultured cells under different conditions and prepared cell lysates for the assay. We evaluated levels of USP22 in cell lines of QBC939, HCCC, RBE, Huh28 and HuCCT1 **(A),** siRNA knockdown efficacy by transfecting 3 different pairs of siRNAs into RBE **(B)**, shRNA knockdown in RBE and QBC939 **(C)**, and overexpression in HCCC and Huh28 **(D)**.

**Supplementary Figure 3. FACS analysis of E-cadherin and vimentin expression in response to USP22 regulation in CCA cell lines.**

**(A-L)** Cell lines of HCCC-EV/HCCC-USP22, Huh28-EV/Huh28-USP22, and RBE-EV/RBE-shUSP22, QBC939-EV/QBC939-shUSP22 were respectively seeded at 1x10^7^ in 6-well plates. After 48 hours, they were dissociated and fixed before staining with E-cadherin or vimentin antibodies prior to FACS analysis. All data are shown as mean ± SD (* p < 0.05, n = 3).

**Supplementary Figure 4. USP22 regulates acetylation of TAK1 and Akt.**

Cell lysates of RBE-shUSP22, HCCC-USP22 and their control were prepared for immunoprecipitation with antibodies to TAK1 or Akt or IgG control. They were analyzed by immunoblotting with antibodies against pan-acetylation, USP22 and GAPDH. Data shown are representative 3 independent experiments.

**Supplementary Figure 5. SIRT1 downregulated E-cadherin and upregulated vimentin in USP22-silenced RBE and QBC939 cell lines.**

**A-F.** RBE-shControl, RBE-shUSP22, RBE-shUSP22+EV and RBE-shUSP22+SIRT1 or QBC939-shControl, QBC939-shUSP22, QBC939-shUSP22+EV and QBC939-shUSP22+SIRT1 were cultured and prepared for analysis of E-cadherin and vimentin expression by FACS as for Supplementary Figure 3 (* p < 0.05, n = 3).

**Supplementary Figure 6. Correlation of USP22 and SIRT1 mRNA expression in CCA tissue samples.**

Analysis of USP22 and SIRT1 mRNA levels in 36 CCA samples by DESeq2 in the TCGA dataset showed no significant correlation between them.

**Supplementary Table 1. Immunohistochemical analysis of protein expression of USP22 and SIRT1**

Protein levels of correlation between USP22 and SIRT1 expression in a set of 57 iCCA in 2nd Hospital of Dalian Medical University, China

| **SIRT1 expression** | **USP22 expression** | | **Total** | ***p*** |
| --- | --- | --- | --- | --- |
|  | **Low** | **High** |  |  |
| **Low** | 24 | 7 | 31 | **0.001** |
| **High** | 8 | 18 | 26 |  |
| **Total** | 32 | 25 |  |  |
