# Supplementary material for: Operative ubiquitin-specific protease 22 deubiquitination confers a more invasive phenotype to cholangiocarcinoma
Source: Cell Death Dis. 2021 Jul 5;12(7):678. doi: 10.1038/s41419-021-03940-0 (PMC8257691; doi:10.1038/s41419-021-03940-0)
Supplement: Supplementary file 2 — Supplementary methods [file 41419_2021_3940_MOESM2_ESM.docx]

**SUPPLEMENTAL INFORMATION**

Operative ubiquitin-specific protease 22 deubiquitination confers a more invasive phenotype to cholangiocarcinoma

Yu Tian, Bo Tang, Chengye Wang, Yan Wang, Jiakai Mao, Yifan Yao, Zhenming Gao, Rui Liang, Mingliang Ye, Shijie Cai, Liming Wang

**Supplementary Materials and Methods**

**Sequences of siRNA or shRNA**

| **Name** | **Sequences (5’ to 3’)** |
| --- | --- |
| SiRNA-USP22#1 | GCAGCUUCAAGGUGGAC AATT |
| SiRNA-USP22#2 | GGAGAAAGAUCACCUCGAATT |
| SiRNA-USP22#3 | GCAUCAUAGACCAGAUCUUTT |
| siControl | UUCUCCGAACGUG- UCACGUTT |
| shRNA-USP22 | GCAUCAUAGACCAGAUCUUTT |
| shRNA-SIRT1 | CCAAGCAGCUAAGAGUAAUTT |
| shControl | UUCUCCGAACGUG- UCACGUTT |

**Antibodies**

| **Name** | **Manufacturer** | **Dilution** | **Catalog number** |
| --- | --- | --- | --- |
| USP22 | Abcam | 1:1000 (WB), 1:100 (IHC),  1:40 (IP) | ab195289 |
| E-cadherin | Abcam | 1:1000 (WB), 1:200 (IF),  1:100 (FACS) | ab1416 |
| Vimentin | Abcam | 1:1000 (WB), 1:200 (IF),  1:100 (FACS) | ab92547 |
| ERK1/2 | Abcam | 1:1000 (WB) | ab184699 |
| p-ERK1/2 | Abcam | 1:1000 (WB) | ab201015 |
| Akt | Abcam | 1:1000 (WB) | ab185633 |
| Bcl-2 | Abcam | 1:1000 (WB) | ab182858 |
| Bax | Abcam | 1:1000 (WB) | ab32503 |
| p-Akt | Abcam | 1:1000 (WB) | ab192623 |
| SIRT1 | Proteintech | 1:1000 (WB), 1μg (IP) | 13161-1-AP |
| Myc-tag | Proteintech | 1:1000 (WB), 1μg (IP) | 9e1 |
| Flag-tag | Proteintech | 1:1000 (WB), 1μg (IP) | 20543-1-AP |
| Ubiquitin | Proteintech | 1:1000 (WB), 1μg (IP) | 10201-2-AP |
| Pan-Acetylation | Proteintech | 1:1000 (WB), 1μg (IP) | 66289-1-Ig |
| GAPDH | Proteintech | 1:1000 (WB) | 60004-1-Ig |
| Goat anti-rabbit | Thermo | 1:5000 (WB) | 31430 |
| Goat anti-mouse  Goat anti-rabbit  Goat anti-mouse | Thermo  Thermo  Thermo | 1:5000 (WB)  1:500 (FACS), 2 µg/mL (IF)  1:500 (FACS), 2 µg/mL (IF) | 31430  F-2765  A28175 |
