# Supplementary figures and images for: Operative ubiquitin-specific protease 22 deubiquitination confers a more invasive phenotype to cholangiocarcinoma
Source: Cell Death Dis. 2021 Jul 5;12(7):678. doi: 10.1038/s41419-021-03940-0 (PMC8257691; doi:10.1038/s41419-021-03940-0)

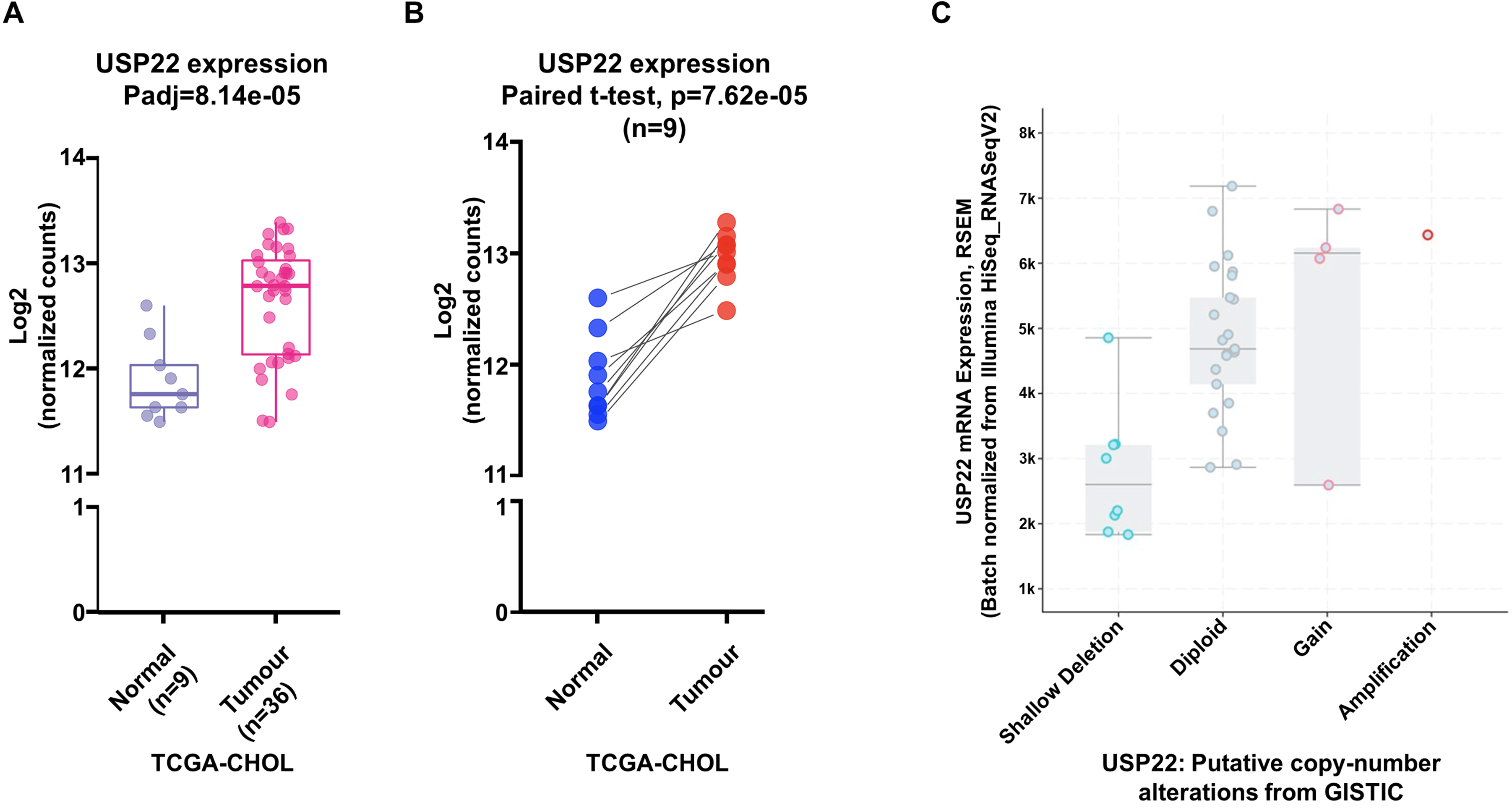

Supplement: Supplementary file 3 — Supplementary Fig. 1 [file 41419_2021_3940_MOESM3_ESM.tif]

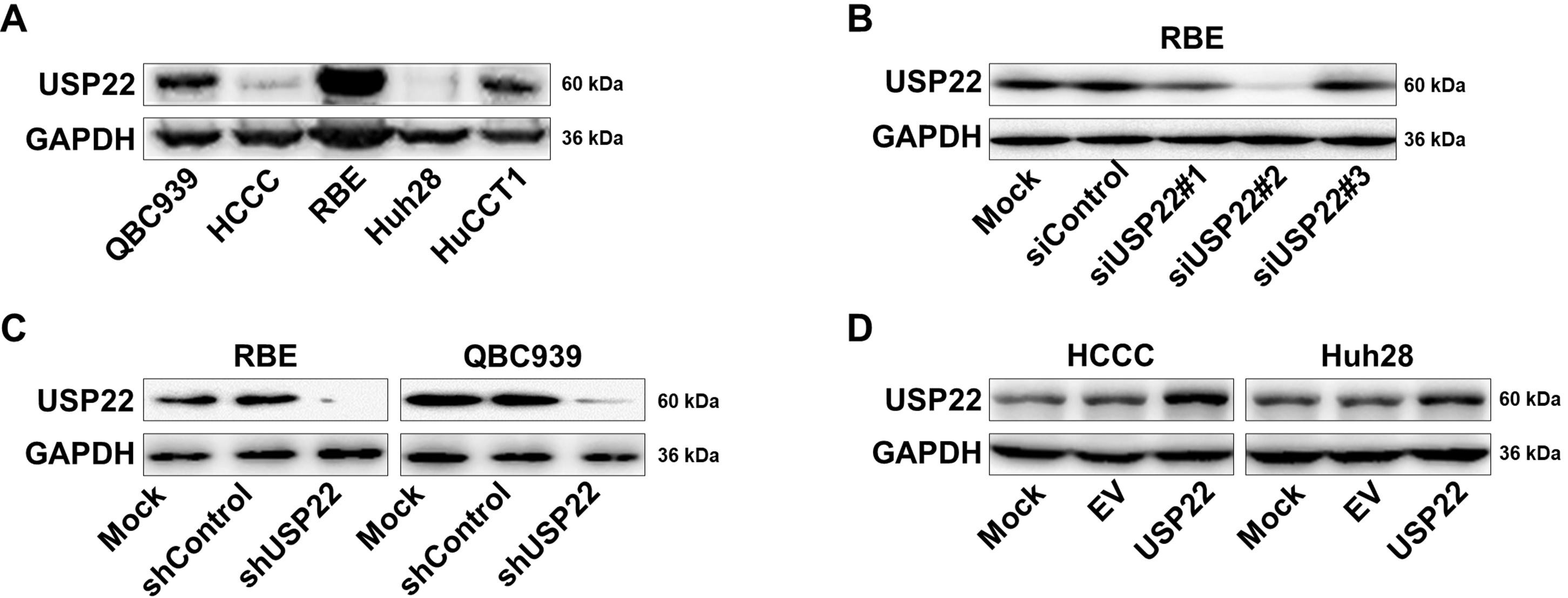

Supplement: Supplementary file 4 — Supplementary Fig. 2 [file 41419_2021_3940_MOESM4_ESM.tif]

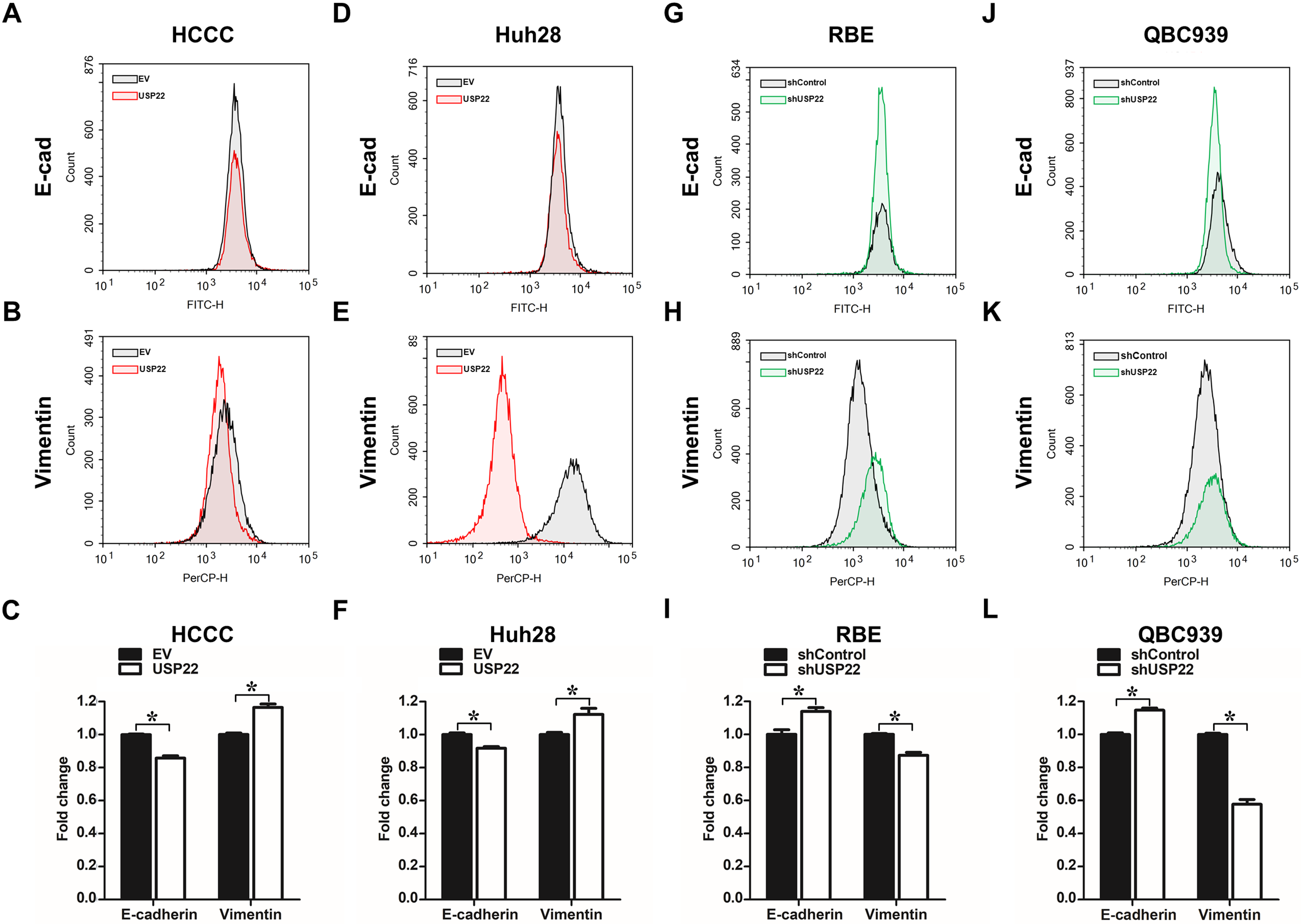

Supplement: Supplementary file 5 — Supplementary Fig. 3 [file 41419_2021_3940_MOESM5_ESM.tif]

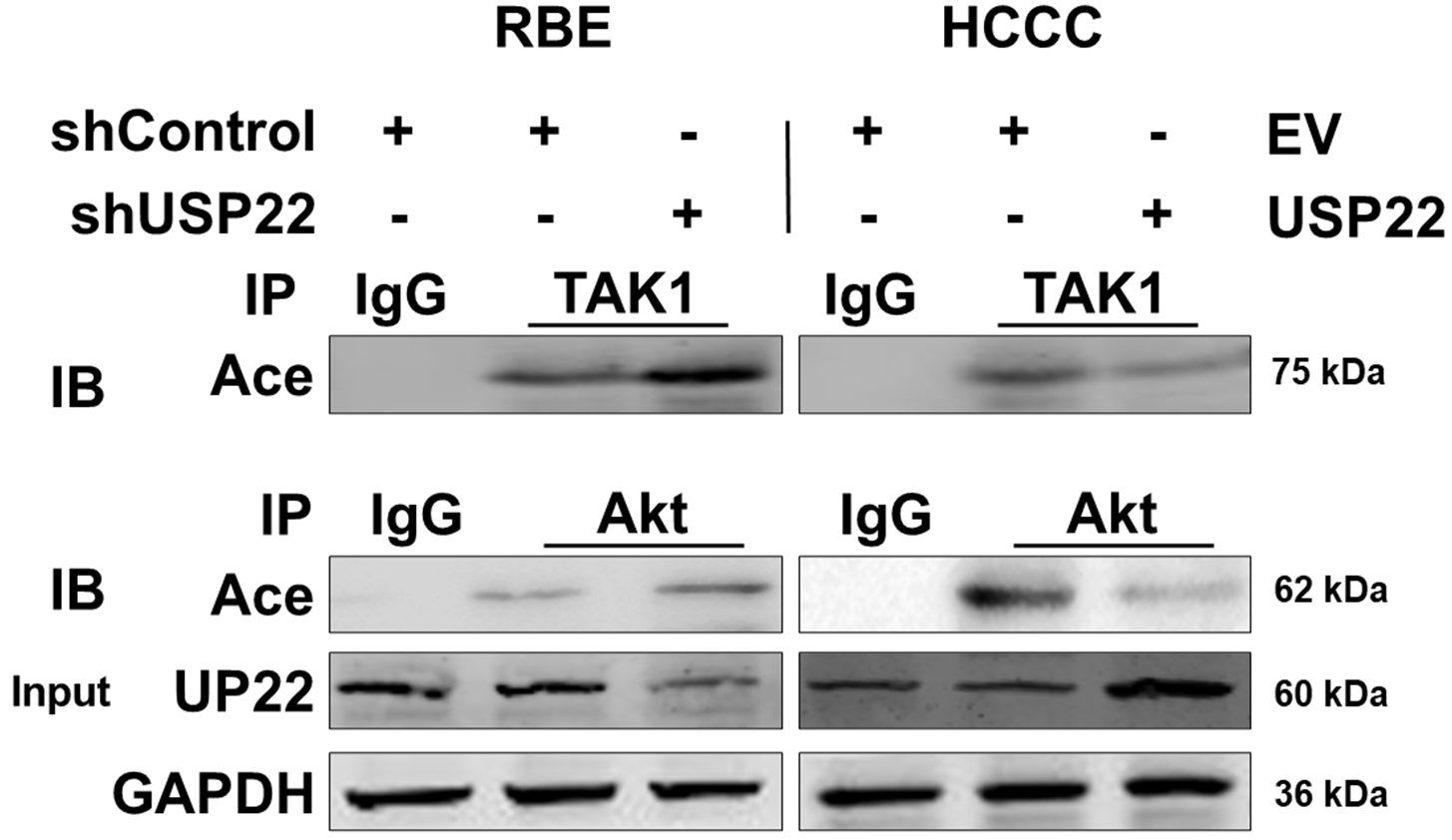

Supplement: Supplementary file 6 — Supplementary Fig. 4 [file 41419_2021_3940_MOESM6_ESM.tif]

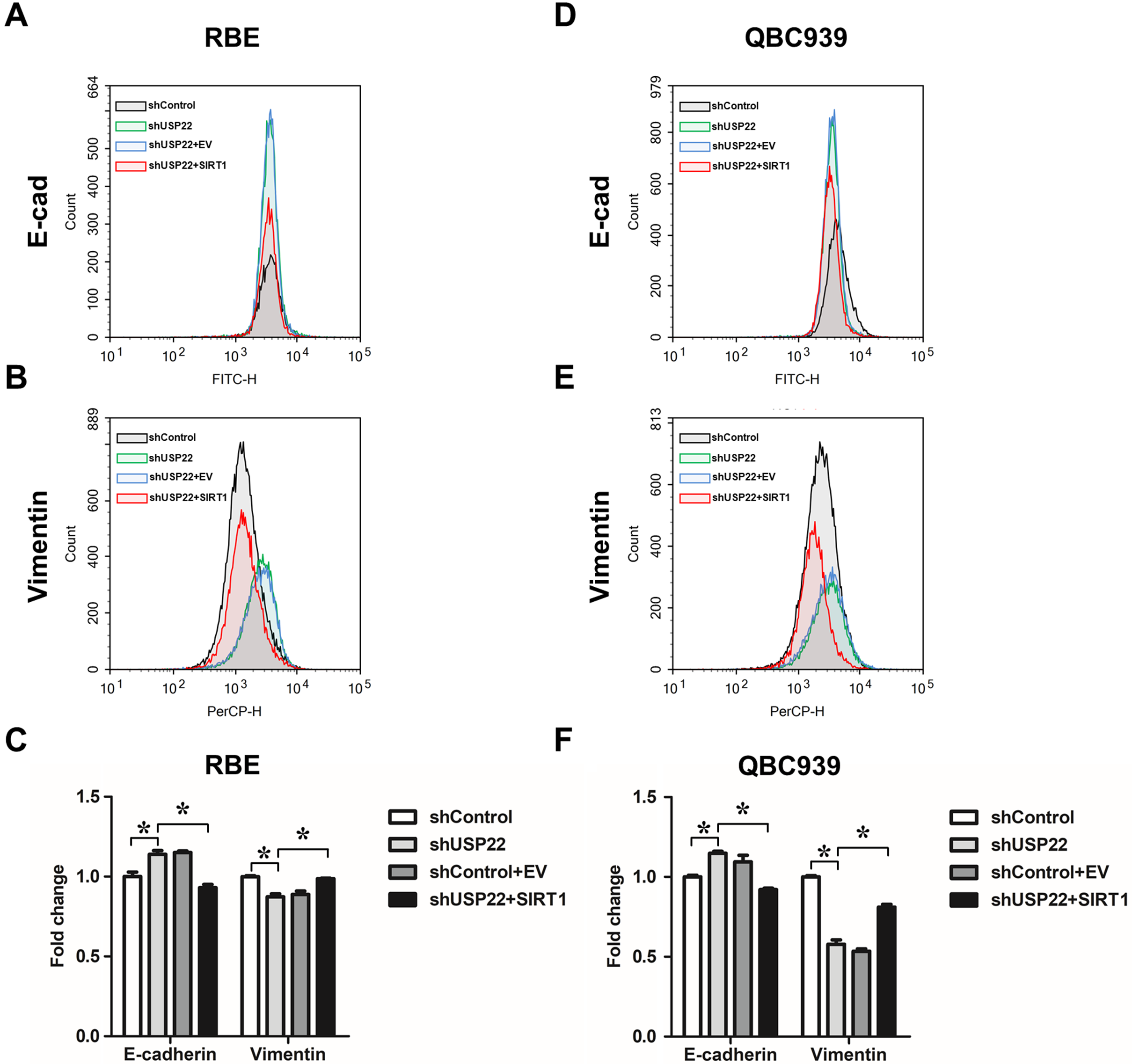

Supplement: Supplementary file 7 — Supplementary Fig. 5 [file 41419_2021_3940_MOESM7_ESM.tif]

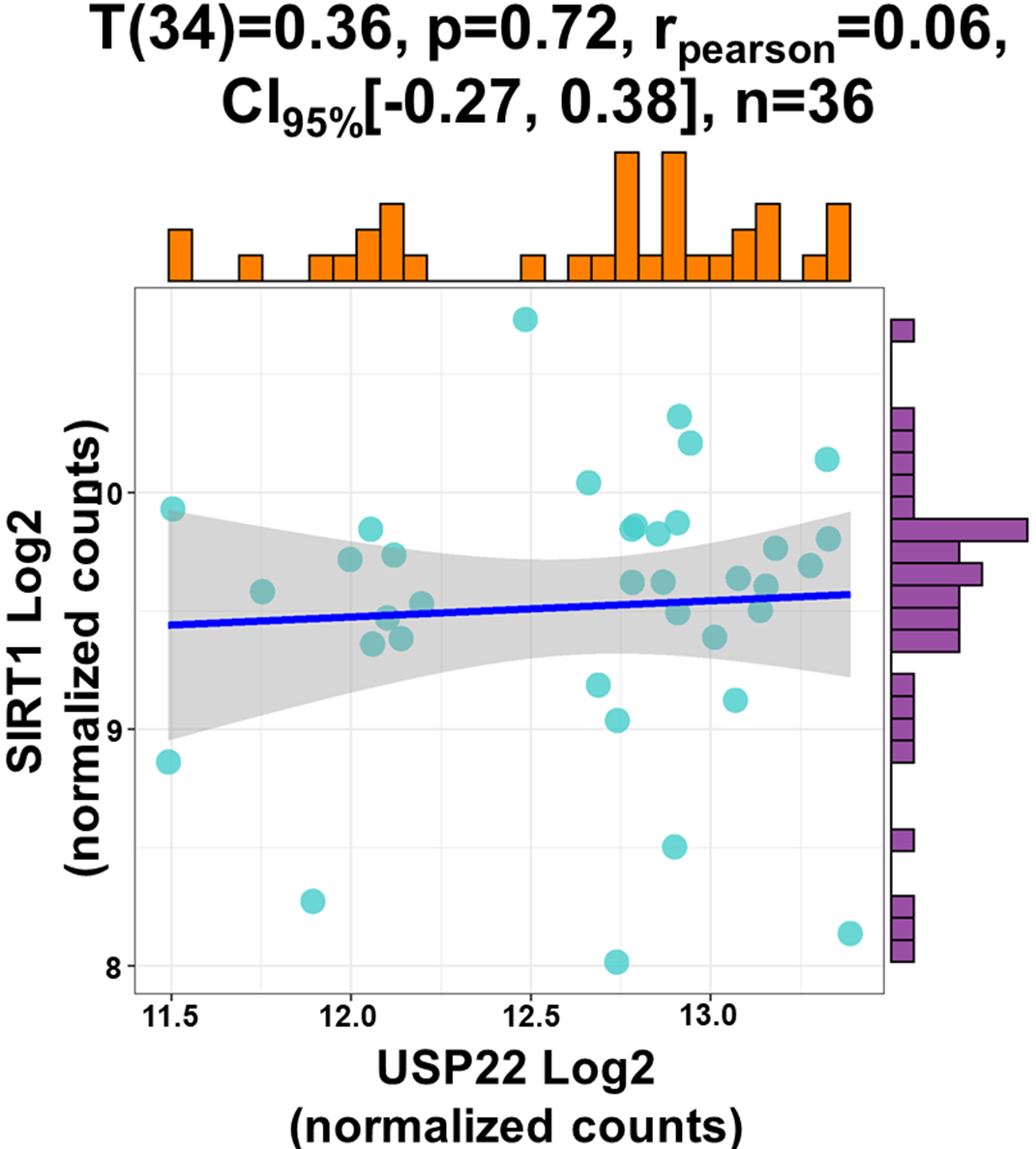

Supplement: Supplementary file 8 — Supplementary Fig. 6 [file 41419_2021_3940_MOESM8_ESM.tif]
